# Novel Visual Nasogastric Tube Insertion System: A Feasibility and Efficiency Study in a Manikin

**DOI:** 10.1155/2016/7532172

**Published:** 2016-11-23

**Authors:** Qiaoya Li, Juan Xie, Jinxing Wu, Rui Guo, Wenwen Ma, Gang Xu, Min Yang, Huisheng Deng

**Affiliations:** ^1^Department of Geriatrics, The First Affiliated Hospital of Chongqing Medical University, Chongqing 400016, China; ^2^Department of Respiratory Medicine, The First Affiliated Hospital of Chongqing Medical University, Chongqing 400016, China; ^3^Department of Critical Care Medicine, The First Affiliated Hospital of Chongqing Medical University, Chongqing 400016, China

## Abstract

*Background*. Conventional nasogastric tube placement is an essential clinical procedure; however, complications may arise from blind manipulation. We tested the feasibility and efficiency of a visual nasogastric tube insertion system (VNGS) using a manikin.* Methods*. A microimaging fiber (0.8 mm) was integrated into the nasogastric tube to create the VNGS. Twenty inexperienced physicians were enrolled and assigned to the visual or conventional group. Each physician performed 10 repeated nasogastric tube insertions with visual guidance or the conventional method; another 20 inexperienced medical students received nasogastric tube insertion training using visual guidance or the conventional method.* Results*. The nasogastric tube successfully reached the stomach and the narrow anatomic structures were visualized with the VNGS. Time required for insertion was significantly shorter in the visual group compared to the conventional group (22.56 ± 3.08 versus 37.30 ± 4.12 seconds, *P* < 0.001). Tube misplacement was observed in 19/100 cases (19%) in the conventional group; no misplacement was observed in the visual group. Less mucosal damage was noted in the visual group (3.43 ± 1.63 versus 9.86 ± 2.31 cm^2^). Medical students performed better NGT insertions (shorter insertion time and less procedure-related complications) after undergoing the visual guidance training.* Conclusions*. The VNGS may provide a new technique for nasogastric tube insertion applicable to clinical use or simulation training.

## 1. Introduction

Nasogastric tube (NGT) insertion is commonly performed in clinical practice for gastric decompression or enteral nutrition [1, 2]. Insertion of a nasogastric tube is relatively safe; however, unintentional misplacement of the nasogastric tubes into the respiratory tract is not uncommon [3–10], and if unrecognized, such misplacement instances can lead to serious consequences, including pneumonia, pneumothorax, atelectasis, bronchopleural fistula, emphysema, and even death [3–9]. Anatomic abnormalities, anesthesia, tracheotomy, or an absent gag reflex can increase the risk of nasogastric tube misplacement. Additionally, because of the potential need for repeated insertions or prolonged procedure times, nasogastric tube insertion is associated with numerous complications, such as hypertension, tachycardia, arrhythmia, mucosal bleeding, intracranial placement, and aortoesophageal fistula [9, 10].

To maximize the insertion efficiency and minimize iatrogenic complications compared to blind NGT insertion, we must identify the most accurate and sustainable methods of prevention. In our previous experiments, a microimaging fiber (0.8 mm) was integrated into a triple-lumen catheter to perform visual sputum suctioning [11, 12]. In addition, we also inserted a 0.8 mm microimaging fiber into an 18 G needle to guide pericardiocentesis and a 14 G needle to guide visual needle cricothyroidotomy [13, 14]. Because of the advantages provided by the small fiber diameter, we integrated the microimaging fiber into a nasogastric tube to guide nasogastric tube insertion. In the current study, the efficiency and feasibility of nasogastric tube insertion with our video-assisted system were evaluated in a human analog model to test the applicability of this method for clinical use. An additional objective was to assess whether the skill of medical students at nasogastric tube insertion could be improved by simulation training with the visual insertion system using a manikin.

## 2. Materials and Methods

### 2.1. Visual Nasogastric Tube Insertion System

**Figure 1:**
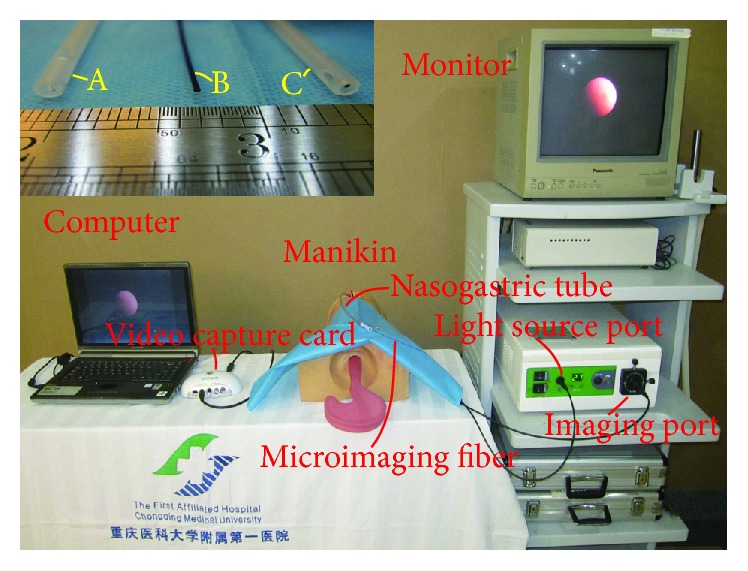
Laboratory human analog model test setup for the visual nasogastric tube insertion system (VNGS). A: nasogastric tube (outer diameter: 4 mm). B: microimaging fiber (outer diameter: 0.8 mm). C: microimaging fiber inserted into the nasogastric tube.

Figure 1 shows the prototype of the visual nasogastric tube insertion system (VNGS). The system consists of an optical fiber (FVS-001MI, Blade, Beijing, China; resolution: 6,000 pixels; outer diameter: 0.8 mm) in a nasogastric tube (12 Fr, Jiangsu Yongning Medical Devices, Yangzhou, Jiangsu, China; outer diameter: 4 mm), a computer monitor, and a processor. The total length of the microimaging fiber was 240 cm. To allow for deep insertion, the length of the working portion was designed to be 650 mm. Depending on the position of the light source, real-time images of the insertion process were recorded by the optical fiber. The signals were then processed and displayed on the computer monitor.

### 2.2. Insertion Tests with a Human Analog Model

In this study, 20 inexperienced physicians were enrolled and randomly divided equally into two groups: the visual group and the conventional group. In both groups, each inexperienced physician was required to perform 10 repeated nasogastric tube insertions in a randomized order using either the visual technique (visual group) or the conventional method (conventional group). The interval time between each performance was one day. In this study, the procedure-related complications mainly consisted of mucosal damage and nasogastric tube misplacement. All inexperienced physicians had graduated less than three months prior to study enrollment, and all had learned NGT insertion when they were medical students, but all inexperienced physicians had performed less than 3 NGT placements. This study was conducted in the animal laboratory of The First Affiliated Hospital of Chongqing Medical University, Chongqing, China.

A human analog model (Zhonghong Teaching Equipment, Shanghai, China) consisting of tongue, nasopharynx, oropharynx, laryngopharynx, trachea, esophagus, and stomach was used. The stomach of this human analog model contains simulated gastric fluid. To facilitate observing and recording mucosal damage during nasogastric tube insertion, the inner surface of the nasal cavity, pharynx, esophagus, and stomach were uniformly painted with red dye to simulate the mucosa. The experiment was conducted according to the following two experimental protocols.

*(1) Nasogastric Tube Insertion with Visual Guidance*. The 0.8 mm microimaging fiber was positioned in the nasogastric tube throughout the entire insertion procedure. Using real-time guidance, the nasogastric tube was advanced through the nostril, visually identifying anatomic structures, until it reached the stomach (see Video 1 in Supplementary Material available online at http://dx.doi.org/10.1155/2016/7532172). Under these conditions, the duration of insertion was defined as the time when the tube touched the nasal cavity until the video demonstrated that the tube was successfully inserted into the stomach. The procedure time was measured anonymously by a single investigator.

*(2) Nasogastric Tube Insertion with Conventional Methods*. The nasogastric tube was blindly inserted following the standard procedure. To monitor any potential damage during NGT insertion, the 0.8 mm microimaging fiber was also delivered into a nasogastric tube but operators were “blind” to the video information. In this group, the duration of insertion was defined as the time when the tube initially touched the nasal cavity until it was successfully inserted in the stomach as determined by operator considering that he/she has inserted the tube into the stomach. The entire insertion procedure was simultaneously recorded for our analysis.

### 2.3. Examination for Mucosa Damage Examination and Misplacement

After the nasogastric tube insertion was completed, the nasal cavity, nasopharynx, oropharynx, laryngopharynx, esophagus, and stomach were exposed when the human analog model was opened. When the insertion was completed, the red dye was found to be damaged to different degrees. To analyze the degree of mucosal damage in the visual conventional groups, we took a picture of the inner surfaces of these anatomical structures using a camera that was affixed to a static support and positioned at a standardized distance. The damaged areas were then quantified using Image J software (National Institute of Health, Bethesda, Maryland, USA), which was able to easily discern different colors in the picture and analyze the damaged areas [15, 16]. After the operators completed the nasogastric tube insertion, we evaluated whether the nasogastric tube was misplaced or not using the real-time images of the VNGS.

### 2.4. Insertion Simulator Training with the Two Methods

Subsequently, 20 medical students were enrolled and also randomly divided equally into a visual group and a conventional group. None of the medical students had previous insertion experience. A 40 min lecture on nasogastric tube insertion, standardized educational videos, and a 15 min demonstration were provided to the participants. Then, each participant practiced nasogastric tube insertion on a human analog model three times once every 3 days using either the visual technique (visual group) or the conventional method (conventional group). After 12 days, the participants from the two groups were asked to repeatedly perform an insertion 10 times using the conventional method and completion times recorded as before. After the learning process, the participants in both groups were asked to independently complete an anonymous questionnaire. The students were asked to record the “usefulness or helpfulness” of the insertion simulator training with the visual guidance or the conventional method based on a survey with a five-point Likert Scale that included the question of whether the visual guidance training or conventional training method was helpful or useful for inserting a nasogastric tube into the stomach (one point = strongly disagree, two points = disagree, three points = neither disagree nor agree, four points = agree, and five points = strongly agree). Data were collected anonymously by a single investigator.

### 2.5. Statistical Analysis

All data were analyzed using SPSS software (Version 21.0, SPSS Ltd., Chicago, Illinois, USA), and normal distribution of parameters was determined using the Shapiro-Wilk method. Normally distributed variables were described by using a mean ± standard deviation (SD) and nonnormally distributed variables were described by the median and interquartile range (IQR). Comparisons of the insertion times and damaged mucosal areas between the conventional and visual groups were analyzed using a two-sample *t*-test. The complication rates related to nasogastric tube insertion between the groups were analyzed with the Pearson Chi-Square test. A *P* value less than 0.05 was considered statistically significant.

## 3. Results

### 3.1. Procedure-Related Complications

**Figure 2:**
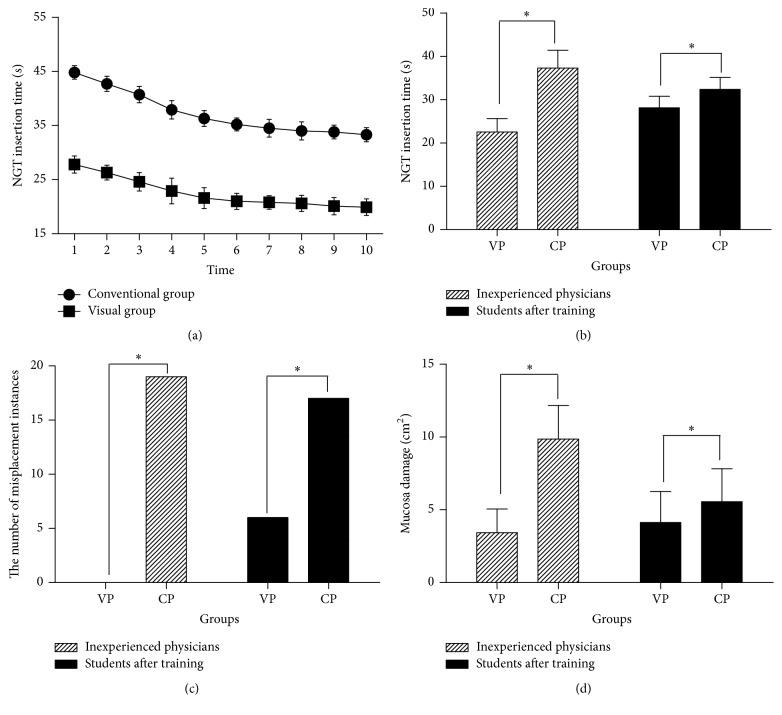
(a) Graph showing the nasogastric tube insertion times of the visual group (VP) and conventional group (CP) of inexperienced physicians with repeated measurements. (b) Comparison of nasogastric tube insertion times. (c) Comparison of nasogastric tube misplacement. (d) Comparison of damaged mucosal areas (*∗* indicates *P* < 0.05).

In the visual group, the nasogastric tube was successfully inserted by all inexperienced physicians. However, in the conventional group, the nasogastric tube was misplaced in 19/100 cases (19%, Figure 2(c)). As shown in Figure 2(d), we confirmed that the use of the VNGS helped with the insertion of the tube into the stomach and produced less mucosal damage (3.43 ± 1.63 versus 9.86 ± 2.31 cm^2^, *P* < 0.001).

### 3.2. Comparison of Nasogastric Tube Insertion Time between the Visual Group and the Conventional Group by Inexperienced Physicians

The procedure time was then compared between the visual group and the conventional group of inexperienced physicians using a two-sample *t*-test.  Figure 2(a) shows the learning curves of both groups. The insertion time of the visual group was 22.56 ± 3.08 seconds, which was significantly less than that of the conventional group (37.30 ± 4.12 seconds, *P* < 0.001, Figure 2(b)).

### 3.3. Visually Guided Nasogastric Tube Insertion

**Figure 3:**
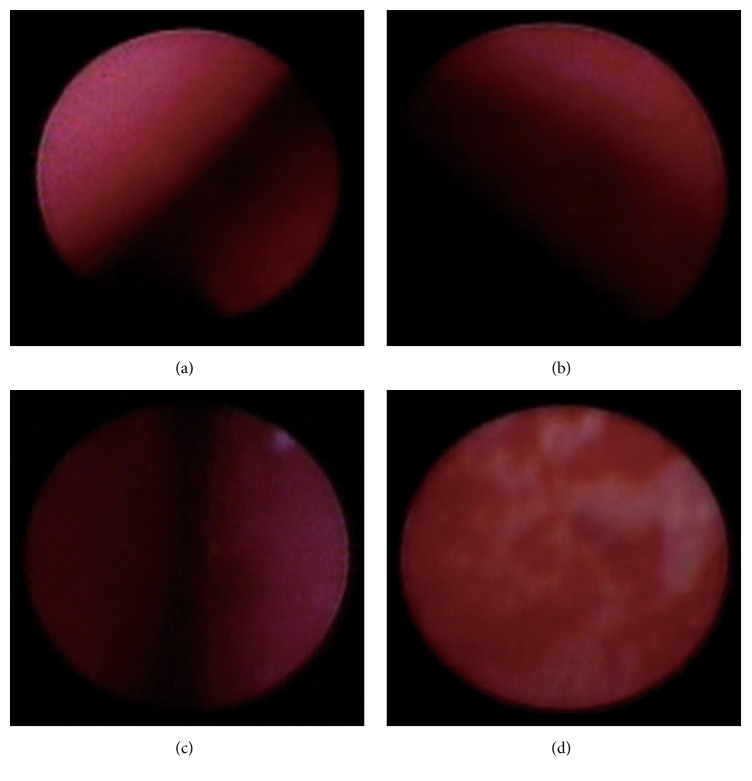
Images from the visual nasogastric tube insertion system (VNGS) in the manikin. (a) Turbinate. (b) Pharynx. (c) Esophageal stenosis. (d) Mucosal damage.

Using real-time guidance, the nasogastric tube was advanced into the nasal cavity and through the narrow anatomical structures (e.g., anatomic stenosis between the turbinates and anatomic stenosis of esophagus) and then inserted into the stomach, which allowed us to acquire information on the anatomic structures (Figure 3, Video 1). This new technique could help inexperienced physicians determine how to properly insert a nasogastric tube.

### 3.4. NGT Insertion Simulator Training

After 12 days of training, 20 medical students were instructed to blindly perform an insertion 10 times.  Figure 2 illustrates that there were fewer complications (misplacement rate: 6% versus 17%, *P* < 0.001, Figure 2(c); mucosal damage: 4.13 ± 2.13 versus 5.56 ± 2.26 cm^2^, *P* < 0.001, Figure 2(d)) and shorter procedure times (28.16 ± 2.67 versus 32.30 ± 2.52 seconds, *P* < 0.001, Figure 2(b)) in the visual group than in the conventional group. In addition, participants reported that the visual guidance training was more helpful or useful for improving their NGT insertion skill level according to the anonymous survey (4.20 ± 1.03 versus 3.20 ± 0.92 points, *P* = 0.034, *P* < 0.05, five-point Likert Scale).

## 4. Discussion

The main findings of this study are that (1) real-time imaging guidance reduces the time required for nasogastric tube insertion and the number of procedure-related complications and (2) insertion simulator training with the VNGS could help inexperienced physicians improve their nasogastric tube insertion skill level.

Nasogastric tube insertion is commonly associated with lung or esophageal misplacement. Tubes often bend and coil in the pyriform sinus and arytenoid cartilage during insertion [17, 18]. In addition, physiological or pathological variations of a patient's functional anatomy can predispose to prolonged procedure or misplacement [19–21]. However, imaging guidance can help operators insert the NGT into the stomach more smoothly because the operator can visualize key anatomical structures (Figure 3, Video 1). In this study, the efficiency and safety of insertion were determined according to the time required for the procedure and the number of complications. Future research should use animal models or clinical trials to determine whether the VNGS improves safety or efficiency.

This human analog model experiment showed that the visual group was more efficient and safer than the conventional group (see Figure 2). In this study, the visual group required less time to perform nasogastric tube insertion than the conventional group, which may have been related to increased familiarity with the anatomic structures provided by the VNGS. Therefore, the NGT insertion technique of the visual group was more efficient. With regard to the safety of NGT insertion, tube misplacement was observed in 19 cases in the conventional group, whereas no misplacement instances were observed in the visual group. Additionally, less mucosal damage was observed in the visual group than in the conventional group.

Nasogastric tube position is an important consideration, and the National Patient Safety Agency has issued guidelines recommending that the nasogastric tube position should be verified before every feeding. Nasogastric tube misplacement is assessed by clinical signs (e.g., coughing, respiratory distress, and tachypnea), auscultation, gastric fluid aspiration, pH measurement, and chest X-ray [22–24], and although these methods may help ascertain whether a nasogastric tube is misplaced, their accuracy should be evaluated in future research. In our study, the nasogastric tube position was easily confirmed with our imaging guidance method, which successfully avoided misplacement.

Although the laryngoscope was developed to assist with nasogastric tube insertion [25–27], it cannot be advanced into the esophagus or deeper structures, thus increasing the difficulty of guiding nasogastric tubes into those structures without mucosal damage or other complications. NGT tube insertion should be performed by experienced physicians to prevent secondary discomfort or tissue damage that can potentially affect the patients' quality of life. The small diameter of microimaging fibers allows for their insertion into a nasogastric tube, and anatomical structures can then be imaged in real time (Figure 3) to assist with insertion or location, thereby minimizing secondary harm to the patient. Additionally, we could easily check the nasogastric tube position before every feeding by delivering the microfiber into the positioned NGT. Therefore, the VNGS may provide an alternative strategy for monitoring nasogastric tube insertion.

During the nasogastric tube insertion, time is an important consideration that is used to evaluate insertion efficiency. In the present research, under similar conditions (e.g., level of operator experience, human analog models, and successful procedure time definitions), the visual group required less time to perform nasogastric tube insertion than the conventional group, which may have been related to the operator's ability to simultaneously visualize the tube location using real-time images provided by the VNGS. Compared with conventional nasogastric tube insertion, this new strategy can simultaneously provide additional information on the anatomic structures of the nasopharynx, oropharynx, laryngopharynx, esophagus, and stomach (Figure 3), which can be used to quickly determine the cause of dysphagia and may provide an early diagnosis for certain asymptomatic diseases.

Simulation technologies for NGT insertion are widely accepted for use in educational applications [28–31]. The present research also evaluated whether training in nasogastric tube insertion with visual guidance versus blind insertions could result in different perceptions of insertions or skill acquisition in NGT insertion. After the medical students had received the same lectures, standardized educational videos, and demonstrations on insertion, they each took insertion simulator training according to different protocols. After different types of training, students were then asked to take a nasogastric tube insertion test, and the procedure time, procedure-related complications, and self-reported questionnaires were recorded. In the NGT insertion test, medical students from the visual group required less time to perform nasogastric tube insertions than the conventional group. In addition, the occurrence of nasogastric tube misplacement and mucosal damage were lower in the visual group. In short, the visual group demonstrated improved performance compared to the conventional group. In response to the self-reported posttraining questionnaire, participants in the visual group expressed a positive attitude toward the visual guidance nasogastric tube insertion. Therefore, the real-time anatomic visualization of the structures provided by the VNGS provided positive benefits to the medical students by improving their understanding of nasogastric tube insertion, which may have led to higher performance scores on the insertion tests.

Overall, our results suggested that the procedure time for nasogastric tube insertion was shorter and the procedure-related complications were lower with real-time imaging guidance. The insertion simulation training with our visual nasogastric tube insertion system (VNGS) could help inexperienced physicians improve their skills in nasogastric tube insertion. Therefore, the additional effort and costs required to train medical students using the VGNS may be a worthwhile investment.

## 5. Limitations

Although the VNGS has many potential benefits, certain problems were observed that must be resolved. The length of the working portion should be designed to be long enough to visualize deeper anatomic structures. This study used a manikin to evaluate the VNGS, and the feasibility and efficiency of the visual nasogastric tube insertion system should be evaluated in animal models or real patients in future studies. Moreover, additional participants and repeated procedures should be included in future studies for a more detailed evaluation of this new system.

## 6. Conclusions

Nasogastric tube insertion with the VNGS was feasible and could possibly decrease procedure duration and procedure-related complications. Therefore, this system may provide a new technique for nasogastric tube insertion simulation training and has the potential for use in clinical applications.

## Supplementary Material

A video (Video 1) showing how the nasogastric tube was delivered into the stomach with our micro-imaging guidance (VNGS).
